# An Extradural Cervical Mass Mimicking Myofascial Neck Pain in an Elderly Female: A Case Report

**DOI:** 10.7759/cureus.90068

**Published:** 2025-08-14

**Authors:** Iustin Scobercea, Kebereab Feyissa, Stephen J Despins

**Affiliations:** 1 Osteopathic Manipulative Medicine, Liberty University College of Osteopathic Medicine, Lynchburg, USA; 2 Sports Medicine/Physical Medicine and Rehabilitation, Collaborative Health Partners Specialty Services, Lynchburg, USA

**Keywords:** chronic pain management, interventional pain medicine, meningioma, osteopathic manipulative therapy, physical medicine and rehabilitation, sports medicine

## Abstract

We detail the case of an 85‑year‑old woman with osteopenia and a history of breast cancer, who came to the Physical Medicine and Rehabilitation (PM&R) clinic complaining of ongoing neck pain without motor, sensory, or neurological deficits. After conservative management failed to provide the patient relief, magnetic resonance imaging revealed an extradural mass at the C4-C6 levels. Neurosurgical evaluation determined that the mass was likely a meningioma. Considering the patient’s age, stable clinical condition, and her personal preferences, she chose to proceed with conservative management, declining both biopsy and surgical intervention. This case report aims to emphasize the importance of patient autonomy, the need for appropriate escalation of care in cases of persistent neck pain, and the principles of functional improvement and quality of life central to PM&R.

## Introduction

Neck pain is a common complaint in older adults. Some estimates rate the prevalence at up to 50% for individuals over the age of 65 [1]. While most cases can be attributed to age-related degenerative changes, persistent or unusual symptoms that resist conservative care warrant deeper investigation [2]. 

Although uncommon, spinal mass lesions, including schwannomas and meningiomas, should be considered in the differential diagnosis of refractory neck pain. Tumors of the spine account for only about 0.5% of all causes of neck and back pain, with cervical spinal tumors comprising a small subset of these cases [3,4]. Schwannomas, which arise from Schwann cells of peripheral nerves, account for approximately 25% of primary spinal tumors and are most commonly found in the thoracic and cervical regions [4]. Meningiomas, by contrast, arise from the meninges and are the most common benign intracranial tumors in adults, accounting for approximately one-third of all primary brain and central nervous system tumors [5]. 

Based on magnetic resonance imaging (MRI) and clinical presentation, the mass in this case is believed to be a meningioma. Meningiomas are more common in middle-aged to older adults, with a higher prevalence in females [4]. Their classification ranges from World Health Organization Grade I (benign) to Grade III (malignant), with 80-85% falling in the Grade I classification [5]. They are slow-growing tumors that can remain asymptomatic for a long time [4]. However, when symptomatic, meningiomas typically present with unilateral radicular pain, motor deficits, and paresthesia [4]. Overall, neurological deficits vary depending on the tumor’s location and the degree of compression [4]. 

Diagnosis of a meningioma is primarily done by MRI with contrast [6]. It typically shows a round, sharply demarcated space-occupying lesion that can exhibit a “dural tail” sign [6], which symbolizes the thickened, enhanced portion of the dura mater that is adjacent to the meningioma. Treatment of a meningioma generally involves surgical resection and adjuvant radiotherapy for particularly malignant cases [6]. 

Here, we present a case of a suspected cervical meningioma that presented as persistent neck and shoulder pain in an elderly woman, managed conservatively in line with physical medicine and rehabilitation principles, which prioritize functional preservation, quality of life, and patient autonomy.

## Case presentation

An 85‑year‑old female with osteopenia and a history of left‑sided breast cancer with mastectomy and right periscapular flap reconstruction, presented to the physical medicine and rehabilitation clinic with a one-year history of right-sided neck pain described as an intense ache that radiated down her right shoulder. On osteopathic examination, we noted tissue texture changes and decreased passive range of motion of her right cervical spine. There was hypertonicity and trigger points palpated along her right cervical paraspinal muscles, medial scapular border, upper trapezius, and levator scapulae. Neurologic exam, including strength, sensation, reflexes, and pulses, was normal. We suspected osteoarthritis of the cervical spine and attributed her symptoms as possibly myofascial in origin and treated her with conservative management that included Tylenol 1000 mg TID PRN, diclofenac gel PRN, heat, ice, massage, aquatic therapy, and dry needling as needed. Furthermore, she was advised to continue her current exercises with physical therapy and treated with osteopathic manipulative treatment of the cervical and thoracic spine. She was advised to follow up in two to three weeks to reassess her symptoms. 

On follow-up, she reported that her symptoms were unchanged from her initial visit despite adequate adherence to her conservative management regimen. She reported that she awakened nightly with pain related to her shoulder and reported no new neurological changes in her symptoms. On examination, multiple trigger points were identified in her right upper trapezius, levator scapula, and rhomboid. Trigger point injections were performed in these areas using a combination of triamcinolone acetonide and lidocaine. At her follow-up, she reported two to three days of pain relief from the injections, but the pain had returned and was worse than before. She reported feeling electric shock-like sensations in her neck and shoulder at times and pain that was worse with neck movements. She was treated with ultrasound-guided perineural injections using dextrose 5% in water (D5W) to the right dorsal rami of C4-C8, the right punctus nervosum (Erb’s point), the right dorsal rami of T1-T3, and the right suprascapular nerve.

**Figure 1:**
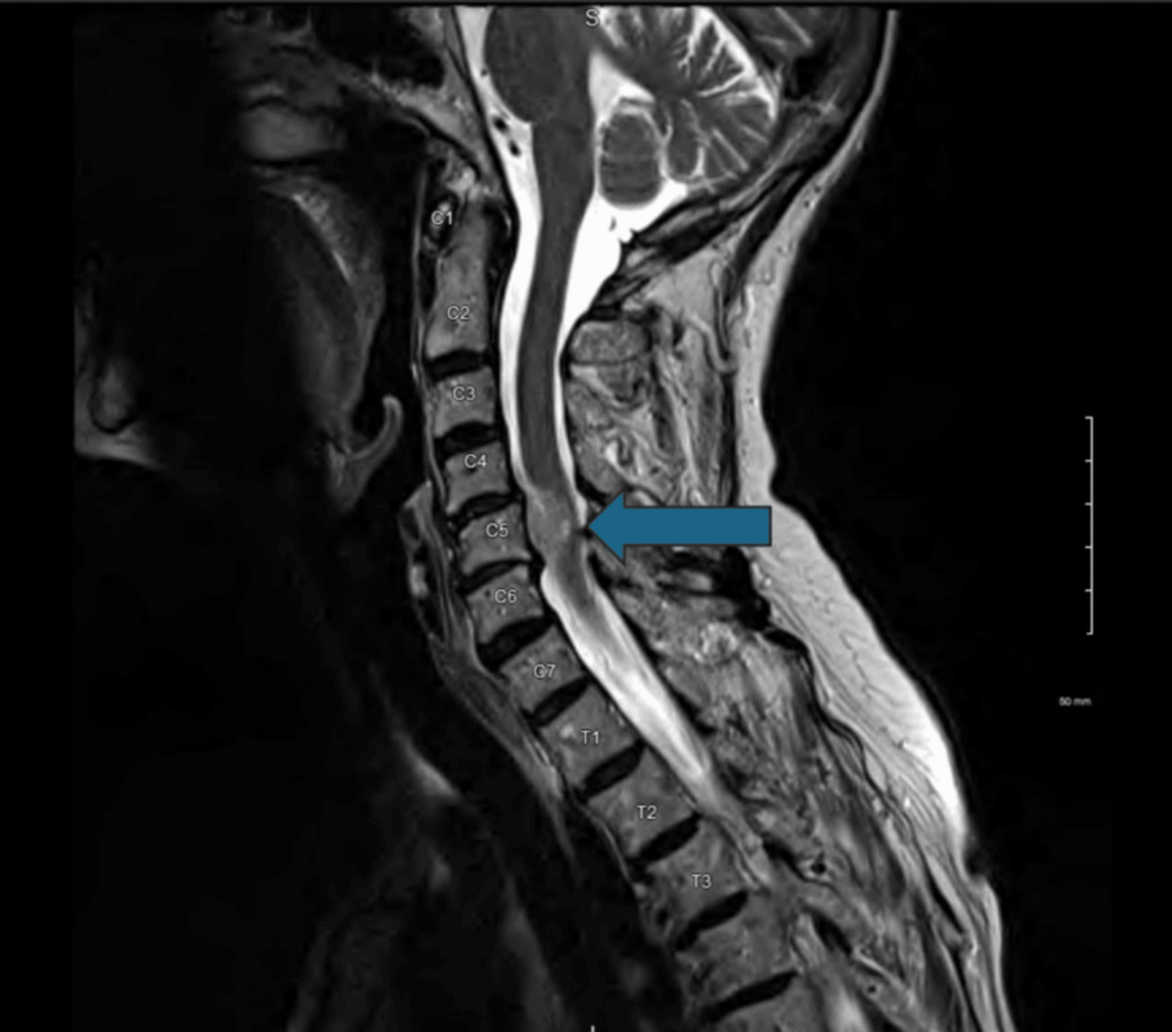
T2-weighted magnetic resonance imaging scan in the sagittal plane showing the suspected meningioma (blue arrow).

Two and a half weeks later, she presented to the clinic for a follow-up. She reported that the D5W injections provided two weeks of pain relief. On exam, there was tenderness along the medial border of the right scapula, right upper trapezius, and right levator scapula. There was also pain and hypertonicity along the lower right cervical and upper right thoracic paraspinal muscles. She again received D5W injections in her cervical spine and right shoulder. At follow-up, it had now been approximately eight weeks since her initial presentation to the clinic, and we assessed that she had failed conservative management (continued Tylenol 1000 mg TID PRN, diclofenac gel PRN, heat, ice, massage, aquatic therapy, and dry needling) for pain, and she was referred for an MRI of her cervical spine, which showed a 0.9 x 1.2 x 1.3 centimeter hyperintense extramedullary and likely extradural mass lesion (Figure 1) centered in the right side of the spinal canal at the C5 vertebral level that displaced the cervical cord to the left with evidence of cord compression. MRI also showed craniocaudal extension of the mass to the adjacent C4-C5 and C5-C6 cervical levels, a “dural tail” (Figure 1), likely consistent with a meningioma.

Despite the results, she reported that she was doing well and denied numbness, tingling, weakness, balance issues, or any other neurological deficits. After discussion, the patient agreed to meet with the neurosurgery team for further evaluation. Her treatment options were explained in detail and included observation, surgical removal, and radiation therapy. She showed good understanding of her situation, and based on the risks and benefits of the treatment options, she decided not to pursue surgery and to manage the mass conservatively. Follow‑up MRI and dynamic X-rays done six months later confirmed no progression or instability.

## Discussion

Management of a cervical mass that presents as chronic neck pain in an 85-year-old female presents a unique challenge due to the need to balance symptom control, treatment risks, and patient quality of life. Furthermore, when considering the treatment spectrum of conservative to surgical, we need to consider factors such as the patient's overall health, the symptoms of the mass, the tumor characteristics, and any potential treatment risks. 

When reviewing the literature, numerous papers [7-11] have presented similar cases of elderly females with a history of persistent neck or cervicobrachial discomfort that is initially managed conservatively but ultimately diagnosed as a cervical mass via magnetic resonance imaging (MRI). In each of these papers [7-11], the patient presented with a variety of neurological deficits, including sensory loss [7], paraparesis [8], upper limb weakness [9], and mild hand weakness [11]. Furthermore, in each paper [7-11], the patient was managed surgically on a spectrum that varied from incomplete surgical resection [7] to complete surgical resection [10] of the mass. Unique to our case is the absence of any significant neurological deficits throughout the patient’s presentation, especially considering the size of the mass that was detailed on MRI. Then, the patient decided to manage the mass conservatively, which is atypical for the presentations detailed in the current literature [7-11]. 

When individuals present with extradural lesions, it is important to rule out the presence of metastasis or hematopoietic disease [10]. Typically, observation is the preferred approach, especially if the mass is slow-growing or asymptomatic [12]. However, if the tumor is growing or causing significant symptoms in the patient, there are two primary treatment options that are considered: a traditional surgical approach or stereotactic radiosurgery (SRS) [10]. A traditional surgical approach can relieve symptoms but is typically more invasive than SRS. Furthermore, it carries risks such as nerve damage, including facial weakness or hearing loss, with some patients experiencing significant balance issues and a marked reduction in independence and quality of life [13]. Conversely, SRS is a non-invasive option that uses focused radiation to stop tumor growth. It typically results in a lower likelihood of nerve damage and is often a preferred treatment approach to preserve quality of life in elderly patients [14]. 

Existing literature shows that the location of a spinal meningioma and whether it is extra- or intradural are important things to consider [10]. Interestingly, the majority of meningiomas, whether intradural or extradural, tend to be in the thoracic spine rather than the cervical spine [10]. It is believed that extradural meningiomas have a worse prognosis due to the possibility of invasion into the dura, making it more difficult to resect completely [10]. However, the literature shows that when considered possible and safe, a total resection should be attempted due to there being an excellent prognosis, with a majority of patients having a resolution of symptoms and no documented tumor recurrence [10]. Subtotal resections are more likely to have a recurrence of the tumor or adverse outcomes [10]. Risks and adverse outcomes associated with surgical resection (regardless if total or subtotal) include tumor recurrence, neurological deficits (i.e., paresis, paraplegia, etc), incomplete resolution of any symptoms that may have been caused by the tumor, and death [10]. It was reasonable in the case of this patient to opt for a watchful approach due to her age, preference to avoid surgery, and absence of neurological deficits, deviating from the existing literature [7-11].

This case highlights the unique challenges of managing an extradural space-occupying mass in an elderly patient. While a conservative approach to management is reasonable given the absence of neurological deficits, the potential for progression of the mass or development of neurological deficits must also be balanced with the invasiveness and risks of surgery in an elderly patient. Moreover, this case reinforces the critical importance of adhering to a standardized, evidence-based escalation of care pathway when patients do not respond to conservative management. In every case reviewed in the literature [7-11], including our own, clinicians appropriately initiated conservative measures and escalated care in a timely, structured manner - incorporating advanced imaging such as MRI and involving neurosurgical consultation when indicated. Failure to follow this systematic approach to escalation risks significant delays in diagnosis and management, which in the context of spinal masses, can result in irreversible neurological deficits, loss of function, and avoidable morbidity and mortality. This highlights the indispensable role of standardized care algorithms in safeguarding patient outcomes, particularly in complex, high-risk clinical scenarios.

## Conclusions

This case highlights the importance of considering spinal mass lesions, including meningiomas, in the differential diagnosis of persistent neck pain unresponsive to conservative management. It demonstrates the value of timely escalation of care through advanced imaging and specialist consultation. This case also underscores the significance of thorough clinical assessment, adherence to a structured diagnostic pathway, and multidisciplinary collaboration in managing complex cervical spine presentations in elderly patients.
